# Case Report: ASI intervention on a child with autism in Saudi Arabia

**DOI:** 10.12688/f1000research.74257.1

**Published:** 2022-01-17

**Authors:** Shahad Alkhalifah, Susan Allen, Hesham Aldhalaan

**Affiliations:** 1 King Faisal Hospital & Research Centre, Centre for Autism Research, Riyadh, 11211, Saudi Arabia; 2 Department of Psychology and Clinical Language Science, University of Reading, Reading, RG6 6UR, UK

**Keywords:** Autism, sensory integration, occupational therapy, Saudi Arabia

## Abstract

**Background**: Ayres Sensory Integration (ASI) is widely employed by occupational therapists working with clients who experience challenges in sensory integration, including those with autism spectrum disorder (ASD). However, there is a dearth of research examining the feasibility of ASI outside of Western nations. This study documented the barriers associated with ASI in Saudi Arabia and assessed whether the intervention could improve process and participation skills.

**Methods**: A pre-test/post-test case study design was used. The participant was a 4-year-old girl with ASD from Saudi Arabia. Data were gathered on sensory processing, motor skills, and participation in activities of daily living. The study used semi-structured interviews and assessments (Sensory Integration and Praxis Tests, the Sensory Processing Measure-Preschool, and the Peabody Developmental Motor Scale-2) to develop goals, identify outcome measures, and plan an ASI intervention.

**Results**: Despite the limited availability of resources (e.g., toys, treatment spaces) and Arabic measures, improvements were observed on motor and sensory tasks and in occupational performance.

**Conclusion**: ASI that adheres to the ASI fidelity tool can be of value for Saudi Arabian children with ASD. Additionally, the study provides a stepping-stone to further research for occupational therapists in Saudi Arabia working with children with ASD.

## Introduction

Sensory integration (SI) is a neurobiological process for finding, assimilating, organizing, and employing sensory information, which helps individuals to interact with the world ( Parham *et al.*, 2011). SI involves sensory perception and sensory reactivity. Sensory perception identifies the quality of sensory input to provide meaning; for example, recognizing an object’s shape or size without using vision. Sensory reactivity is the ability to organize and regulate one’s responses to sensory information ( Schaaf & Mailloux, 2015). SI challenges are common among people with autism spectrum disorder (ASD) ( Tomchek & Koenig, 2016). According to the American Psychiatric Association’s DSM-5 guidelines (2013), hyperreactivity and hyporeactivity to sensory input are features of ASD. Such issues can cause poor concentration and sensory over-reaction ( Jorquera-Cabrera *et al.*, 2017). Al-Heizan *et al.* (2015) reported that 84.8% of children in their ASD sample in Saudi Arabia (SA) had definite sensory processing challenges. However, such challenges can be difficult to identify and may be overlooked, especially by occupational therapists (OT) not trained in SI ( Al-Heizan *et al.*, 2015).

The existing literature shows that services for children with ASD are underdeveloped in SA ( Alkhalifah & Aldhalaan, 2018). The Saudi government has recently encouraged healthcare professionals to train in Western countries to acquire evidence-based, up-to-date information concerning ASD interventions ( Alnemary *et al.*, 2016). Alshehri *et al.* (2019) found that Saudi OT practitioners are frustrated by limited resources; the lack of Arabic assessment tools, materials, and insufficient clinical knowledge about intervention protocols act as barriers to evidence-based practice ( Alkhalifah, 2019).

The Ayres Sensory Integration ^®^ (ASI) intervention utilizes “individually tailored sensory–motor activities contextualized in play at the just-right challenge to promote adaptive responses and foster functional skills as a foundation for participation in occupations” ( Schaaf *et al.*, 2018, p.1). International research suggests that ASI is an evidence-based way to improve communication, social interaction, cognitive, academic/pre-academic, adaptive/self-help, behavioural, and motor skills in children aged 4-12 years with ASD ( Schoen *et al.*, 2019).

SA has a unique culture, and there has been little exploration of whether existing SI interventions and measures, primarily developed in Western contexts, are suitable for its population ( Al-Heizan *et al.*, 2015). The differences between Arab and Western countries could affect interpretations of SI ( Alkhakifah, 2019). For instance, Arabs “tend to interact with a direct body orientation, stand close together, touch frequently, and demonstrate unique use of paralinguistics” ( Al-Khateeb *et al.*, 2014, p.240). On the low energy/weak and movement sensitivity items of the Short Sensory Profile, more Australian children with ASD scored in the typical range than Saudi children with ASD ( Al-Heizan *et al.*, 2015). Parenting culture in SA is protective, possibly impacting children’s opportunities for motor, proprioceptive, and/or vestibular development ( Al-Heizan *et al.*, 2015). Alnemary *et al.*’s review (2017) indicated that, in the context of ASD, there has been little research on either the services available or treatment outcomes in Arab countries. With 167,000 Saudis estimated to have ASD ( Alnemary *et al.*, 2017), there is a clear need for OT services ( Alshehri *et al.*, 2019) to examine the effectiveness of treatment options, such as manual ASI. The rationale for the current study is to contribute towards filling these gaps in the research in the context of SA.

### Objectives

Before assessing the efficacy of OT-ASI in SA, it is necessary to examine such an intervention’s feasibility when applied to individuals and to address implementation obstacles ( Portney & Watkins, 2000). While previous studies based on children with impaired SI suggest that OT-ASI can improve SI and occupational performance, such studies did not focus on Arab countries ( Schaaf & Nightlinger, 2007). Moreover, many such studies failed to use replicable protocols ( Schaaf *et al.*, 2018). The current study hypothesized that participation challenges of children with ASD were linked to SI impairments. Therefore, this study had the following objectives: (1) identify barriers associated with providing OT-ASI to a child with ASD in SA and (2) establish the efficacy of this intervention in the SA context. In line with these objectives, this research set out to answer the following question: to what extent is OT-ASI appropriate and effective in the Saudi context?

## Case report

### Case study participant

The inclusion criteria were: a) diagnosis of ASD by a multidisciplinary team using standardized tools, such as the The Autism Diagnostic Observation Schedule (ADOS) ( Lord *et al.*, 2000); b) living in Riyadh; c) parents providing voluntary consent; d) attending a mainstream preschool; e) experiencing challenges with activities of daily living (ADLs); f) aged 4-12 years. Individuals with other medical conditions were excluded. Recruitment was through parent invitation at Riyadh’s Centre in October 2018 at Autism Research (CFAR). The study was presented to CFAR at King Faisal Hospital and ethical approval granted for it to proceed.

One Saudi girl, referred to as L, aged 4 years and 7 months, met the criteria. L was diagnosed with ASD at 2 years and 3 months. She attended school full-time, where she participated in small groups of students with special educational needs. Her teachers had not undertaken ASD-specific training, and she was not receiving ASD-specific interventions. However, she had participated in a 2-month home-based speech intervention, completing 3-6 sessions weekly. While this appeared to improve her communication skills, L’s interactions with her peers and teachers were limited, and she exhibited frequent tantrums during transitions or changes to routine. Despite her poor communication skills, her cognitive development was typical. There was no relevant family medical or social history.

### Step I 

The study was guided by data-driven decision making (DDDM), a systematic process that aids OTs in clinical reasoning while addressing client needs ( Schaaf & Mailloux, 2015). The study involved eight steps.

The first step involved a semi-structured interview with L’s mother, using occupational profiles, to identify how L interacts with her environment ( AOTA, 2017) and her participation strengths and challenges ( Schaaf & Mailloux, 2015). These profiles were translated into Arabic so the mother could understand them. L was reported to not initiate actions independently and often seem clumsy. She was described as a fussy child. She was unable to put on socks and shoes. She struggled to play with her peers and bumped into others, impacting social relationships. She also had difficulty writing. L’s teacher reported that L had a limited attention span and struggled to accept rules and use crayons.

### Step II

In step II assessments were completed to identify factors affecting participation. To assess impairment related to SI and praxis, we used the *Sensory Integration and Praxis Tests* (SIPTs) and the Arabic *Sensory Processing Measure-Preschool* (SPM-P; Centre for Autism Research). The SIPTs comprise 17 individual sub-tests for children aged 4-8 years, measuring neurological ability to integrate the sensory inputs required for coordination, motor planning, visual-spatial actions, and perception ( Schaaf & Lane, 2015). The SIPTs discriminate between normal and dysfunctional children in the United States at a statistically significant level ( Schaaf & Mailloux, 2015). Of the 17 test items, 13 show reliability scores of.70 or more, which indicates heigh reliability. Moreover, the test has high inter-rater reliability due to the detailed scoring and because therapists using the SIPTs undertake training ( Schaaf & Mailloux, 2015). Each SIPT results in a standard score that reflects the child’s performance compared to age-matched norms; the average score for a group of a given age is 0 ( Schaaf & Mailloux, 2015). Scores below -1 standard deviation are considered evidence of dysfunction.

The Arabic SPM-P consists of home and classroom forms, which are suitable for children aged 2-5 years ( Alkhalifah *et al.*, 2020). The home form has 75 items and the classroom form has 62. The home form has excellent internal consistency when used with ASD (α = .93) and typically developing (α = .95) children ( Alkhalifah *et al.*, 2020). The Arabic classroom form has not yet been assessed, though the English version has excellent internal consistency when used with clinical (α = .93) and typical (α = .94) samples ( Jorquera-Cabrera *et al.*, 2017). Both forms use Likert scales to measure sensory behaviour frequency in sensory processing, praxis ability, and social participation ( Alkhalifah *et al.*, 2020; Jorquera-Cabrera *et al.*, 2017). Both were used in this study because assessing children with ASD in different environments gives a comprehensive understanding of SI function ( Jorquera-Cabrera *et al.*, 2017).

**Figure 1.**
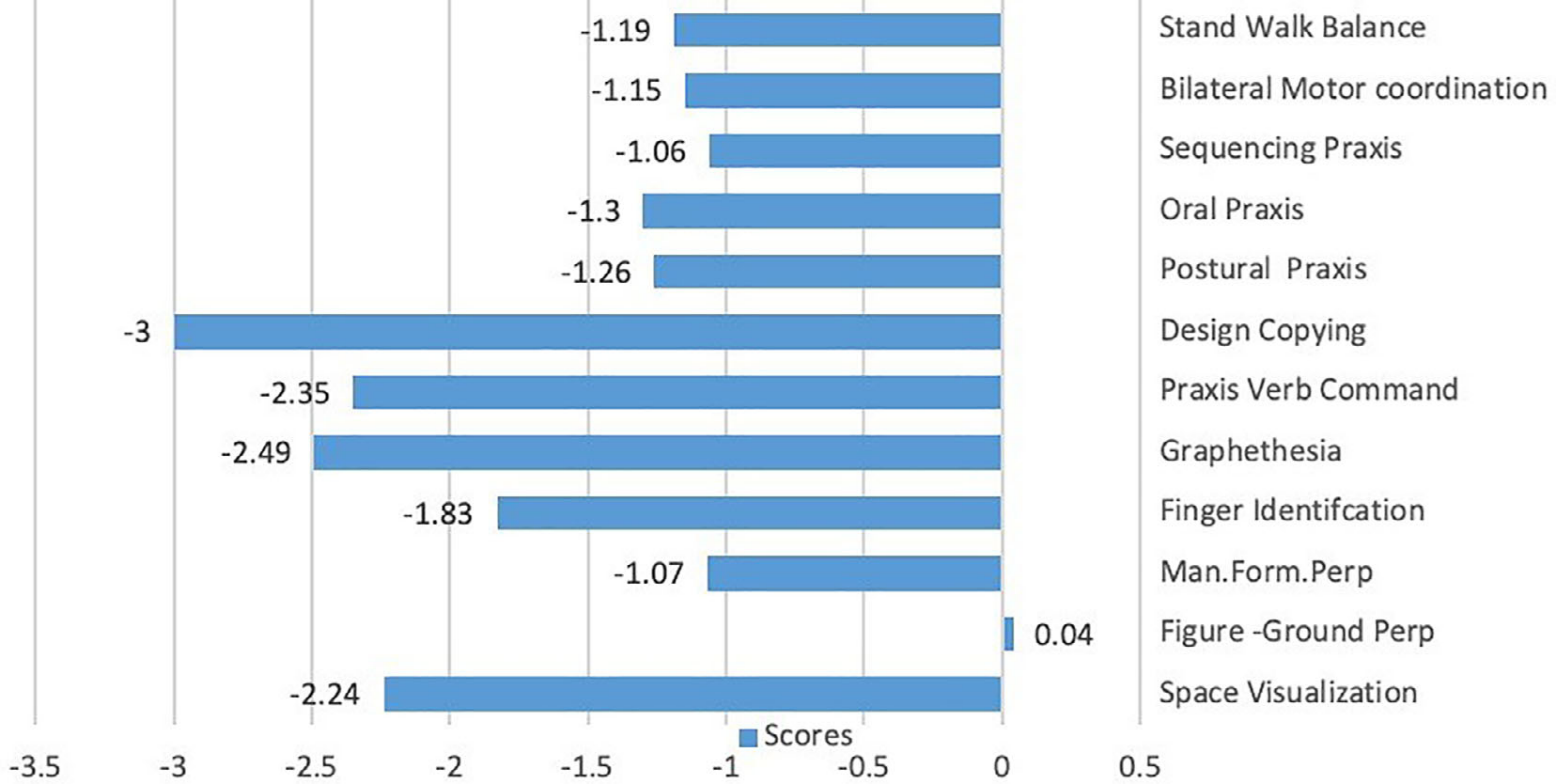
L’s Sensory Integration and Praxis Tests scores.

L attempted 13 of the 17 SIPTs, completing 12 (see Figure 1). Scores indicated difficulty with tactile and kinesthetic processing, especially manual form perception, finger identification, and graphesthesia. L struggled in motor planning as measured by postural praxis, oral praxis, sequencing praxis, design copy, and bilateral motor coordination. Similarly, she scored low for space visualization. L was unable to participate in the localization of tactile stimuli, kinesthesis, and constructional praxis tests, and unable to fully participate in motor accuracy and postrotary nystagmus tests.

The findings from the Arabic SPM-P home and classroom forms are illustrated in Table 1. The environmental difference score demonstrated consistency across environments. Both measures indicated that L struggled with body awareness, balance and motion, social participation, hearing, and touch. The SPM-P revealed problems in planning and idea items and suggested her tactile, body awareness, and auditory systems were hyper-responsive.

Additionally, we used the *Peabody Developmental Motor Scale-2* (PDMS-2), a task-observation test designed to evaluate fine and gross motor skills in children aged 0–5 years ( Folio & Fewell, 2000). We selected the PDMS-2 because L’s challenges were associated with impaired fine and gross motor skills, which are common in ASD ( Provost *et al.*, 2007). Using a motor skills measure that assesses visual-motor integrations was important as visuomotor connections seem to be aberrant in ASD ( Sharer *et al.*, 2015).

L performed very poorly on tests of both fine and gross motor skills on the PDMS-2, and her total motor quotient score was very poor ( Table 3). Additionally, on clinical observation, L was unable to maintain a flexion position for more than 10 seconds, while prone extension was achieved for 3 seconds. She showed difficulty with sequential finger touching and ramped arm movements. She also avoided touch in the absence of vision.

### Step III: Hypothesis Generation

In step III, hypotheses were generated to link the assessment findings to L’s performance on ADLs ( Schaaf & Mailloux, 2015). Overall, the findings suggested L’s participation challenges were due to impairments related to SI and praxis. Specifically, it was hypothesized that L had somatodyspraxia ( Schaaf & Mailloux, 2015).

### Step IV: The Goal Development and Scaling

In step IV, goals were established using the Goal Attainment Scale (GAS; King’s College London) ( Ruble *et al.*, 2012). The GAS is both systematic and sensitive to changes in functioning associated with ASI. It entails describing the individual’s present level of functioning for a given goal and then scaling it for the anticipated level of function over the intervention period. GAS helps assess outcomes that are challenging to measure with traditional instruments ( Mailloux *et al.*, 2007), and is a valid and reliable tool ( Steenbeek *et al.*, 2007) for individuals with ASD ( Ruble *et al.*, 2012). In a recent systematic review of ASI, Schaaf *et al.* (2018) concluded that “GAS is a strong, sensitive, and meaningful outcome” (p. 7). GAS uses a five-point scale, with values ranging from -2 to 2, scaled with equally spaced probability intervals ( Kiresuk *et al.*, 2014). A score of 0 reflects the anticipated level of function, while -1 and -2 suggest a level of attainment less/much less than anticipated, respectively, while +1 and +2 suggest better/much better than anticipated, respectively. Scores for each goal are used to calculate T-scores, representing the extent to which functioning improved to the degree anticipated.

The first author worked with L’s mother to identify specific and measurable goals reflecting L’s functional challenges ( Schaaf & Mailloux, 2015). L’s mother knew about her difficulties in school and could contribute to setting both school- and home-based goals. As recommended by Ruble *et al.* (2012), goals were quality checked by the second author.

L’s goals were:

1. *Dressing*: Improve participation in dressing, to independently put on shoes after verbal prompts with fewer than two physical prompts.
2. *Fine motor skills*: Improve participation in learning activities, to draw or reproduce a circle with two verbal prompts.
3. *Play*: Improve participation in social play, engaging with a peer in age-appropriate activities for 10 minutes with two adult redirections.
4. *Safety:* Improve participation in playtime, navigating the playground without bumping into objects or people.

These goals were based on the premise that improving sensory processing would increase participation in everyday activities ( Schaaf & Mailloux, 2015).

### Step V

In step V, the authors determined the proximal and distal outcomes to use to track progress *.*

*Distal Outcome Measure:* Changes in L’s goals were measured by GAS, enabling the evaluation of progress toward specific, measurable, and time-dependent goals

*Proximal Outcome Measures:* The Arabic SPM-P and the PDMS were used as secondary outcome measures. The former was used to measure sensory reactivity, praxis, and social participation, the latter to measure fine and gross motor skills.

### Step VI

In step VI, the intervention was planned. We agreed that L would participate in 1-hour ASI sessions twice weekly for 10 weeks, meeting her mother in person and her teacher over the phone every week for 30 minutes. Each session would provide sensory-rich experiences to elicit changes in the aberrant SI hypothesized to underlie L’s participation challenges. For therapy room sessions, interventions would consist of a beginning, middle, and end ( Schaaf & Davies, 2010). Schaaf and Davies (2010) emphasized that ASI entails attention to meaningful activity, requiring adaptive responses and active participation from the child. Accordingly, L’s sessions were designed to provide a challenge level that was “just right” for her sensory systems ( Schaaf & Lane, 2015).

### Step VII

In step VII, the intervention was conducted. The first author, a licensed OT with certification in ASI and six years of experience working with children with ASD, delivered the intervention activities tailored to L’s needs. L had numerous opportunities to play with tactile-rich apparatus, to improve her proprioceptive and tactile perception, and praxis. Moreover, through pulling, pushing, and hanging activities, she was encouraged to stretch and engage her muscles. She participated in various active sensory–motor activities. She used a scooter board to experience proprioceptive and vestibular sensations and increase body awareness. She also engaged in jumping into a ball pit, climbing, rolling, and crawling. There were opportunities to change the apparatus and the rhythm, duration, frequency, and/or intensity of sensory experiences, based on L’s responses. In line with ASI principles, the first author and child cultivated an active, trusting relationship, with the former monitoring activity demands to ensure a just-right level of challenge ( Schaaf & Mailloux, 2015).

The use of the ASI Fidelity Measure also ensured the intervention was in line with ASI principles ( Parham *et al.*, 2011). This measure defines the structure of the intervention and process elements ( Parham *et al.*, 2011, 2011; Schaaf & Mailloux, 2015). A score of 80 is a tentative cut-off point for adherence to ASI ( Parham *et al.*, 2011). The measure has high interrater reliability for both total fidelity scores (.98) and individual items (.94–.99). The validity of the measure is strong as raters can distinguish ASI sessions from other interventions with 92% accuracy ( Parham *et al.*, 2011).

All 20 sessions were videotaped, and the second author, trained in the application of the measure and with 30 years of experience working with children with ASD, assessed four randomly selected tapes. Additionally, the second author provided weekly consultations to the first author and was available throughout to discuss intervention challenges. Only one session produced an unacceptable fidelity score, after which the authors collaborated to ensure acceptable fidelity in all other sessions. The mean fidelity score across the four sessions assessed was 85 ( *SD* = 9).

### Step VIII

In step VIII, outcomes were measured and progress monitored, which included the mother’s ratings for each goal and consultation with L’s teacher to calculate a T *-*score using Kiresuk *et al.*’s (2014) formula. Outcomes for the dressing goal (rating = 1) exceeded expectations. Outcomes for the other goals (ratings = 0) were consistent with expectations. L’s post-intervention T-score was 62, suggesting she performed better than expected.

When interviewed six weeks post-intervention, L’s mother described her as happier, more sociable, better able to play safely, and less impulsive. She said:

“ *L has started to play more. Yesterday, she created a restaurant menu and when she was upset, she drew a sad face! She can sit and play for more than 20 minutes. In addition, she hugs me without using too much force. L does not react to unfamiliar tasks with crying anymore! I wish many centres could offer the ASI… L cannot wait for your sessions*!”

When interviewed six weeks post-intervention, L’s teacher said that her *“attention in the classroom was so much better and she is starting to participate in making choices during activities.*” Both reported that L’s fine motor and social skills had improved and she showed fewer difficulties when playing.

The secondary outcomes showed that L improved in various domains. The results for the Arabic SPM-P home and classroom show L improved in sensory reactivity, praxis, and social participation in both home and classroom settings ( Table 1). Similarly, the PDMS-2 data indicated L’s overall motor skills improved ( Table 2). In a conversation six months post-intervention, L’s mother reported that, despite receiving no additional sessions, L’s behaviour at home and school continued to improve.

**Table 1.** Pre- and post-intervention Arabic Sensory Processing Measure-Preschool (SPM-P) school form scale scores.

Area	Pre-intervention				Post-intervention			
	Raw score	T-score	Percentile rank	Interpretation	Raw score	T-score	Percentile rank	Interpretation
Social participation	31	80	99	Some problem	19	54	66	Typical
Vision	17	58	79	Typical	10	40	16	Typical
Hearing	20	70	98	Definitive dysfunction	12	53	62	Typical
Touch	18	68	62	Some problem	12	56	66	Typical
Body awareness	20	74	99	Typical	12	56	69	Typical
Balance & motion	22	72	99	Some problem	11	53	62	Typical
Planning & ideation	27	77	99	Some problem	13	54	66	Typical
Total sensory system	107	69	97	Some problem	64	51	54	Typical

**Table 2.** Pre- and post-intervention Arabic Sensory Processing Measure-Preschool (SPM-P) home form scale scores.

Area	Pre-intervention				Post- intervention			
	Raw score	T-score	Percentile rank	Interpretation	Raw score	T-score	Percentile rank	Interpretation
Social participation	26	66	95	Definite dysfunction	10	44	27	Typical
Vision	12	51	54	Typical	17	58	79	Typical
Hearing	22	74	99	Definite dysfunction	14	60	84	Some problems
Touch	16	66	95	Some problems	16	46	34	Typical
Body awareness	12	55	73	Definite dysfunction	11	50	50	Typical
Balance & motion	14	64	92	Definite dysfunction	13	55	69	Typical
Planning & ideation	20	66	95	Definite dysfunction	9	40	16	Typical
Total sensory system	82	62	88	Some problems	78	56	73	Typical

**Table 3.** Pre- and post-intervention Peabody Developmental Motor Scale-2 (PDMS-2) scores.

	Pre-intervention		Post-intervention	
Variable	Quotient	95% CI	Quotient	95% CI
Fine motor	64	56-72	85	77-93
Gross motor quotient	70	66-74	91	87-95
Total motor quotient	64	58-74	88	82-94

## Discussion

Given the lack of research describing ASI in SA, this case study examined the feasibility and effectiveness of this intervention. The study suggests ASI is feasible within SA and can lead to improvements in individualized functional goals in ADL and performance on sensory and motor tasks.

While OT-ASI in SA appears feasible, its delivery was not straightforward. Since OT-ASI has only recently been introduced to SA, few parents were aware of its potential, making it challenging to find willing participants. This was partially addressed by translating materials that explained what ASI is and why it is valuable, which was a labour-intensive process.

Teachers in SA rarely participate in interventions and have limited knowledge of ASD and sensory issues ( Haimour & Obaidat, 2013). Therefore, the first author spent time explaining to L’s teacher how to complete the Arabic SPM-P and was available to answer questions. Additionally, the first author was available to provide L’s mother and teacher with guidance to understand the intervention. However, therapists in SA working with individuals with ASD and their families often lack time to model interventions or address families’ complex medical and behavioural needs ( Alnemary *et al.*, 2016).

A few Arabic language resources are available, such as a translation of the SPM-P ( Alkhalifah *et al.*, 2020). It is necessary to translate more resources into Arabic to increase understanding of ASD and SI among teachers in SA, to inform their practice and help them identify pupils who might benefit from OT interventions.

According to Serbin *et al.* (2014), a child’s family is the main influence on their health and well-being. However, the parents of disabled children are especially susceptible to stress ( Dempsey *et al.*, 2009), potentially limiting their contributions to GAS. Indeed, L’s mother struggled to imagine goals that would reflect better regulation and participation in family life. Moreover, Saudi mothers often place great trust in healthcare providers and may feel it inappropriate to engage in the therapeutic process ( Alkhalifah & Aldhalaan, 2018); this appeared true of L’s mother, who was hesitant to participate in GAS. Nonetheless, this study suggests that GAS is useful for quantifying individual outcomes ( Mailloux *et al.*, 2007). GAS also enables researchers to measure changes in tailored, functional, and parent-generated goals. Therefore, it is a valuable supplement to other assessments when measuring outcomes associated with individual interventions ( Schaaf & Mailloux, 2015).

The use of proximal and distal goals was helpful in demonstrating links between the underlying challenges in SI and daily occupation. The SIPTs had not previously been applied in an Arab nation, so this was the first exploration of their suitability in SA. However, some SIPTs can be difficult to administer to non-English-speaking children ( Bodison & Mailloux, 2006). Roley *et al.* (2015) highlighted that SI tests can be challenging for children with ASD, with only 63% of their sample finishing most tests. Therefore, proxy measures (e.g., observations) are recommended ( Schaaf & Mailloux, 2015). Other limitations of the SIPTs are that they rely on 40-year-old normative data and their software is incompatible with modern operating systems ( Mailloux *et al.*, 2018). The SIPTs are also only suitable as pre-post-test measures for periods in excess of eight months ( Mailloux *et al.*, 2018). In the current study, L could not complete all of the SIPTs; therefore, additional measures were utilized, with GAS completed post-intervention.

Future related studies should utilize the Evaluation of Ayres Sensory Integration (EASI) tool, to be released in 2020. Suitably qualified therapists will have open access to the EASI, which will incorporate normative data collected internationally. The EASI will meet the demand for the assessment of ASI constructs with psychometrically validated and internationally applicable measurement tools ( Mailloux *et al.*, 2018).

Treatment integrity was confirmed using the Ayres Sensory Integration ^®^ Fidelity Measure ( Parham *et al.*, 2011). This presented some challenges. For instance, it was necessary to videotape the sessions. While L’s mother agreed to this, many Saudi families may not, due to stigma and religious reasons ( Alotaibi & Almalk, 2016). L’s mother expressed concerns and wanted to ensure no one beyond the first author and the training group would see the video; she personally refused to be videoed, or audio recorded, for religious reasons. Furthermore, accessing suitable training was difficult. Some Saudi professionals do not share alternative treatments with families because of the limited hands-on support available for ASD interventions ( Alshehri et al., 2019). This shortage of qualified trainers could reduce the quality of services in SA for ASD ( Alnemary *et al.*, 2016). The first author undertook training abroad for over one year. To complete this study, the therapist required confidence and experience in navigating the local protocol and ethical consent processes ( Schaaf & Nightlinger, 2007).

Neuroscientific evidence suggests OT-ASI interventions elicit increased neurological adaptations if they simultaneously target multiple sensory systems ( Lane & Schaaf, 2010). However, acquiring apparatus suitable for stimulating L’s various sensory systems was challenging; few items were available in SA, so they had to be ordered internationally. The ASI fidelity tool requires a suitable room, points of suspension, and equipment; negotiating additional space and financing for this was time-consuming.

### Preliminary evidence for the efficacy of ASI in SA

Post-intervention, L performed better on sensory and motor tasks and, in the context of ADLs, she demonstrated improvements in fine, gross, and overall motor skills. Previously, her performance was characterized as “very poor”, subsequently it was “average” or “below average”. These findings are in line with the review of Schaaf *et al.* (2018).

Jasmin *et al.* (2009) found that sensorimotor deficits in those with ASD underlie their day-to-day functioning. Congruent with this, L’s improvements in sensory processing and motor skills were reflected in enhanced performance in ADLs. For all tasks identified by GAS, she exhibited increased ability. She improved, as much as anticipated, her capacities to draw, play appropriately, and dress herself, positive changes that were reflected in the comments of L’s mother and teacher.

### Limitations

As the study focused on OT–ASI with one child, the results are limited with respect to generalizability. While case histories only provide level V evidence, they are useful for exploring transitions to different cultures ( Schaaf & Nightlinger, 2007). Another limitation is that the evaluation was completed by the therapist delivering the treatment (not independent assessors).

## Conclusion

This study hypothesized that participation challenges observed in a child with ASD were linked to SI impairments. In support of this, improvements in her participation were seen after an intervention targeting SI. Therefore, this study provides initial evidence that Saudi children with ASD could benefit from ASI treatments and offers insights into the factors affecting delivery.

This study highlights the lack of normative data on SI in SA and a need for further Arabic assessment tools. Moreover, there is little awareness of how individuals with ASD face challenges in SI, and relevant resources are insufficient. The government could address these issues through ASD and ASI workshops, establishing scholarships for training, and committing financial resources to assessment tools and treatment spaces.

Creating videos was particularly valuable as the child’s responses could be reviewed, helping the therapist to reflect. Similarly, the intervention was facilitated by DDDM (Schaaf & Blanche, 2012). Post-graduate training in ASI and GAS supports service delivery and the evaluation of outcomes, mentored training is recommended for newly qualified OTs.

However, the study is limited by only including one participant and by the lack of blinding, issues that future studies should address. Nevertheless, this study provides a stepping-stone to further research in this area for OTs in SA working with children with ASD whose functioning is impacted by aberrant SI.

### Key points for occupational therapy

• There is a need for more data on SI in SA and Arabic assessment tools.
• Government-funded workshops, training scholarships, assessment tools and treatment spaces would help address challenges in SA.
• Post-graduate and mentored training in ASI and GAS is recommended for newly qualified OTs.

#### Ethics and consent

The study was presented to the Centre for Autism Research (CFAR) at King Faisal Hospital and ethical approval was granted for it to proceed. L’s mother gave written informed consent for L to be involved in the study and L’s information to be published in this manuscript. L’s mother also gave permission for L to be recorded during her sessions. All the recorded sessions are stored in CFAR’s system, in a separate, secure (locked) folder to ensure data protection. Further, in accordance with research data management policy, all recorded sessions have been secured in password protected files only accessible to the researchers. The videos will be retained for 10 years, before being securely disposed.

## Data availability

All data underlying the results are available as part of the article and no additional source data are required.

## Grant information

The author(s) declared that no grants were involved in supporting this work.

## Competing interests

No competing interests were disclosed.
