# Case Report: ASI intervention on a child with autism in Saudi Arabia

**DOI:** 10.12688/f1000research.74257.2

**Published:** 2022-05-04

**Authors:** Shahad Alkhalifah, Susan Allen, Hesham Aldhalaan

**Affiliations:** 1 King Faisal Hospital & Research Centre, Centre for Autism Research, Riyadh, 11211, Saudi Arabia; 2 Department of Psychology and Clinical Language Science, University of Reading, Reading, RG6 6UR, UK

**Keywords:** Autism, sensory integration, occupational therapy, Saudi Arabia

## Abstract

**Background**: Ayres Sensory Integration (ASI) is widely employed by occupational therapists working with clients who experience challenges in sensory integration, including those with autism spectrum disorder (ASD). However, there is a dearth of research examining the feasibility of ASI outside of Western nations. This study documented the barriers associated with ASI in Saudi Arabia and assessed whether the intervention could improve process and participation skills.

**Methods**: Single-Subject experimental design was used. The participant was a 4-year-old girl with ASD from Saudi Arabia. Data were gathered on sensory processing, motor skills, and participation in activities of daily living. The study used semi-structured interviews and assessments (Sensory Integration and Praxis Tests, the Sensory Processing Measure-Preschool, and the Peabody Developmental Motor Scale-2) to develop goals, identify outcome measures, and plan an ASI intervention.

**Results**: Despite the limited availability of resources (e.g., toys, treatment spaces) and Arabic measures, improvements were observed on motor and sensory tasks and in occupational performance.

**Conclusion**: ASI that adheres to the ASI fidelity tool can be of value for Saudi Arabian children with ASD. Additionally, the study provides a stepping-stone to further research for occupational therapists in Saudi Arabia working with children with ASD.

## Introduction

Sensory integration (SI) is a neurobiological process for finding, assimilating, organizing, and employing sensory information, which helps individuals to interact with the world ( Parham *et al.*, 2011). SI involves sensory perception and sensory reactivity. Sensory perception identifies the quality of sensory input to provide meaning; for example, recognizing an object’s shape or size without using vision. Sensory reactivity is the ability to organize and regulate one’s responses to sensory information ( Schaaf & Mailloux, 2015). SI challenges are common among people with autism spectrum disorder (ASD) ( Tomchek & Koenig, 2016). According to the American Psychiatric Association’s DSM-5 guidelines (2013), hyperreactivity and hyporeactivity to sensory input are features of ASD. Such issues can cause poor concentration and sensory over-reaction ( Jorquera-Cabrera *et al.*, 2017). Al-Heizan *et al.* (2015) reported that 84.8% of children in their ASD sample in Saudi Arabia (SA) had definite sensory processing challenges. However, such challenges can be difficult to identify and may be overlooked, especially by occupational therapists (OT) not trained in SI ( Al-Heizan *et al.*, 2015).

SA has a unique culture, and there has been little exploration of whether existing SI interventions and measures, primarily developed in Western contexts, are suitable for its population ( Al-Heizan *et al.*, 2015). Alshehri *et al.* (2019) found that Saudi OT practitioners are frustrated by limited resources; the lack of Arabic assessment instruments, materials, and insufficient clinical knowledge about intervention protocols act as barriers to evidence-based practice ( Alkhalifah, 2019). The Saudi government has recently encouraged healthcare professionals to train in Western countries to acquire evidence-based, up-to-date information concerning ASD interventions ( Alnemary *et al.*, 2016).

Yet, the differences between Arab and Western countries could affect interpretations of SI (Alkhakifah, 2019). For instance, Arabs “tend to interact with a direct body orientation, stand close together, touch frequently, and demonstrate unique use of paralinguistics” ( Al Khateeb *et al.*, 2014, p.240). On the low energy/weak and movement sensitivity items of the Short Sensory Profile, more Australian children with ASD scored in the typical range than Saudi children with ASD ( Al-Heizan *et al.*, 2015). There is a culture of protective parenting in SA, possibly impacting children’s opportunities for motor, proprioceptive, and/or vestibular development ( Al-Heizan *et al.*, 2015). Alnemary *et al.*’s review (2017) indicated that, in the context of ASD, there has been little research on either the services available or treatment outcomes in Arab countries. With 167,000 Saudis estimated to have ASD ( Alnemary *et al.*, 2017), there is a clear need for OT services ( Alshehri *et al.*, 2019) to examine the effectiveness of treatment options, such as manual ASI.

The Ayres Sensory Integration ^®^ (ASI) intervention utilizes “individually tailored sensory–motor activities contextualized in play at the just-right challenge to promote adaptive responses and foster functional skills as a foundation for participation in occupations” ( Schaaf *et al.*, 2018, p.1). International research suggests that ASI is an evidence-based way to improve communication, social interaction, cognitive, academic/pre-academic, adaptive/self-help, behavioral, and motor skills in children aged 4-12 years with ASD ( Schoen *et al.*, 2019). However, the existing literature shows that services for children with ASD are underdeveloped in SA ( Alkhalifah & Aldhalaan, 2018). While previous studies based on children with impaired SI suggested that OT-ASI can improve SI and occupational performance, such studies did not focus on Arab countries ( Schaaf & Nightlinger, 2007). Moreover, many such studies failed to use replicable protocols ( Schaaf *et al.*, 2018).

The rationale for the current study is to contribute towards filling these gaps in the research in the context of SA. The current study hypothesized that participation challenges of children with ASD are linked to SI impairments. Therefore, this study had the following objectives: (1) identify barriers associated with providing OT-ASI to a child with ASD in SA and (2) establish the efficacy of this intervention in the SA context. In line with these objectives, this research set out to answer the following question: to what extent is OT-ASI appropriate and effective in the Saudi context?

## Method

An experimental single case methodology was used, whereby an AS intervention was deployed with the aim of improving motor and behavioral patterns in a Saudi child diagnosed with ASD. The single case design has the advantage of clearly demonstrating whether the intervention was effective in one subject ( Dallery & Raiff, 2014). The ABA withdrawal design was chosen to demonstrate the effects of the manipulation of the independent variable (in this case, the AS intervention) by withdrawing the intervention during a second baseline phase and measuring the results with the same instruments as in the preintervention phase ( Byiers *et al.*, 2012). Measuring the outcomes at various times (before, during, and after the intervention) while systematically manipulating the independent variable to test the hypothesis allows for careful experimental evaluation of effects ( Lobo *et al.*, 2017). The experimental dimension also pertains to the objectives and research question, namely, to test the effectiveness of OT-ASI for a Saudi child with ASD. The study was guided by data-driven decision making (DDDM), a systematic process that aids OTs in clinical reasoning while addressing client needs ( Schaaf & Mailloux, 2015). During the first six weekly data collections from December 2018 to January 2019, the authors ensured that the baseline was stable. The intervention consisted of 2 sessions a week, 60 minutes each session, for 10 weeks. Everything concluded with a six-week withdrawal phase in May-June 2019.

To identify the strengths and participation challenges, we reviewed previous medical reports, conducted a preliminary interview with the mother of the child and the schoolteacher, and performed a systematic observation of the child’s behaviour. Pre-test assessments were completed, including the *Sensory Integration and Praxis Tests* (SIPTs; Ayres, 1989), the Arabic *Sensory Processing Measure-Preschool* (SPM-P; Alkhalifah *et al.*, 2020), and the *Peabody Developmental Motor Scale-2* (PDMS-2; Folio & Fewell, 2000). Based on assessment data, the hypothesis was generated, and specific goals were developed.

The intervention was documented carefully, and the ASI Fidelity Measure ensured that the intervention was in line with ASI principles ( Parham *et al.*, 2011). The first author, a licensed OT with certification in ASI and six years of experience working with children with ASD, delivered the intervention activities tailored to the participant’s needs. All 20 sessions were videotaped, and the second author, trained in the application of the measure and with 30 years of experience working with children with ASD, assessed four randomly selected tapes.

The post-intervention assessment was conducted to gain insight into the child’s progress and how successful the implementation of OT-ASI was in meeting her needs. Additionally, post-intervention parent and teacher interviews were conducted to evaluate the reports of progress. Clinical observations about the new outcomes were made and the evidence for the efficacy of OT-ASI in SA was discussed.

### Participant

Several exclusion criteria were applied to identify suitable participants. From the families with ASD children that came to OT consultations, we first selected a ten of patients that would be suitable for ASI intervention with sensory issues. Secondly, children with other medical conditions were excluded. Thirdly, children that were not enrolled in mainstream preschool were excluded. We contacted all the remaining families, explained to them the scope and purpose of the research, and invited them to take part in the study. Only one family agreed to participate and committed to the lengthy intervention. The other six families we contacted did not agree to be recorded or were not willing to commit to such a long intervention.

L, a Saudi girl aged 4 years and 7 months, was diagnosed with level-2 ASD at 2 years and 3 months by a multidisciplinary team based on DSM-5 ASD criteria. Her parents provided voluntary consent to participate in the study at Riyadh’s Centre for Autism Research (CFAR) in October 2018. The study was presented to CFAR at King Faisal Hospital and ethical approval was granted for it to proceed.

Her mother provided a detailed history, revealing that the girl was born after a full-term pregnancy with no birth complications. L’s medical reports showed no sign of early infancy complications either and her overall health was good. There was no other relevant family medical or social history.

A semi-structured interview, using occupational profiles, was conducted with L’s mother to identify how L interacts with her environment ( AOTA, 2017) and to identify her participation strengths and challenges ( Schaaf & Mailloux, 2015). These profiles were translated into Arabic so the mother could understand them.

L had participated in a 2-month home-based speech intervention, completing 3-6 sessions weekly. While this appeared to improve her communication skills, L’s interactions with her peers were limited, and she exhibited frequent tantrums during transitions or changes to routine. Despite her poor communication skills, her cognitive development was typical. In the semi-structured interview, her mother reported that L can follow simplified instructions, especially when given visual cues. She described L as a fast learner, who loves to sing and play with building blocks. The mother was concerned since L found it difficult to make choices and was fussy, always saying no. She was unable to put on socks and shoes and seemed unsure of how to play with other children. She also had difficulties with drawing and with linking it to writing.

L attended school full-time in Riyadh, where she participated in small groups of students with special educational needs. Her teachers had not undertaken ASD-specific training, and she was not receiving ASD-specific interventions. The teacher reported that she used to bump into peers excessively on the playground, avoided activities involving motor skills, and presented difficulties in holding or using crayons or other objects. L’s teacher reported that L had a limited attention span and struggled to accept rules. All these challenges were reported to negatively impacted her social relationships. participation in the classroom, and learning development.

### Assessment

To assess impairment related to SI and praxis, we used the *Sensory Integration and Praxis Tests* (SIPTs) and the Arabic *Sensory Processing Measure-Preschool* (SPM-P). The SIPTs comprise 17 individual sub-tests for children aged 4-8 years, measuring neurological ability to integrate the sensory inputs required for coordination, motor planning, visual-spatial actions, and perception ( Schaaf & Lane, 2015). The SIPTs discriminate between normal and dysfunctional children in the United States at a statistically significant level ( Schaaf & Mailloux, 2015). Considering that an acceptable reliability score for research purposes is .70, completing 13 out of 17 test items means that the reliability threshold could be reached around that figure. Each SIPT results in a standard score that reflects the child’s performance compared to age-matched norms; the average score for a group of given age is 0 ( Schaaf & Mailloux, 2015). Scores below -1 standard deviation are considered evidence of dysfunction.

The Arabic SPM-P consists of home and classroom forms, which are suitable for children aged 2-5 years ( Alkhalifah *et al.*, 2020). The home form has 75 items, and the classroom form has 62. The home form has excellent internal consistency when used with ASD (α = .93) and typically developing (α = .95) children ( Alkhalifah *et al.*, 2020). The Arabic classroom form has not yet been assessed, though the English version has excellent internal consistency when used with clinical (α = .93) and typical (α = .94) samples (Parham & Ecker, 2007). Both forms use Likert scales to measure sensory behavior frequency in sensory processing, praxis ability, and social participation (Parham & Ecker, 2007). Both were used in this study because assessing children with ASD in different environments gives a comprehensive understanding of SI function (Parham & Ecker, 2007).

Additionally, we used the *Peabody Developmental Motor Scale-2* (PDMS-2), a task-observation test designed to evaluate fine and gross motor skills in children aged 0–5 years ( Folio & Fewell, 2000). We selected the PDMS-2 because L’s challenges were associated with impaired fine and gross motor skills, which are common in ASD ( Provost *et al.*, 2007). Using a motor skills measure that assesses visual-motor integrations was important as visuomotor connections seem to be aberrant in ASD ( Sharer *et al.*, 2015).

Unstructured clinical observations had been gathered during the assessment period. L's overall performance revealed that she had poor equilibrium reaction. She was using her whole body rather than stabilising her body when playing with a ball. She could not grade the force or time to release of the ball. L demonstrated very poor grasp of objects and it was difficult for her to manipulate them around the playground. Additionally, L was unable to maintain a flexion position for more than 10 seconds, while prone extension was achieved for 3 seconds. She showed difficulty with sequential finger touching and ramped arm movements.

Her emotional stability was dependent on the type of tasks she was performing. For example, during the SIPT and PDMS-2 assessments, she refused or cried when confronted with unfamiliar tasks. Her anxiety generated impulsive behavior and affected her overall attitude towards changes or encounters with unfamiliar objects. Whenever she felt overwhelmed or struggled to overcome an obstacle, she started crying. When the therapist urged her to jump, she was unable to and immediately showed signs of utter frustration. Any changes to rules were perceived as frustrating and she threw tantrums during the assessment whenever she felt uncomfortable with something.

Her reactions to new toys and spontaneous behavior were also poor. The playground overwhelmed her instead of provoking joy and excitement. For instance, when she was presented with a space hopper, which was unfamiliar to her, she did not know what to do, nor did she try to explore it. She had limited coping strategies when things felt out of control, or the activities were perceived as too hard. She avoided or ran away from tasks that seemed unfamiliar, such as swinging back and forth. She would say no to any new attempt and it took some time for the therapist to change her mood and bring her back. She also had difficulties in imitation or copying a new behavior and following simple directions. The therapist’s hypothesis was underlying praxis associated with poor imagination, ideation, imitation.

### Assessment findings

**Figure 1.**
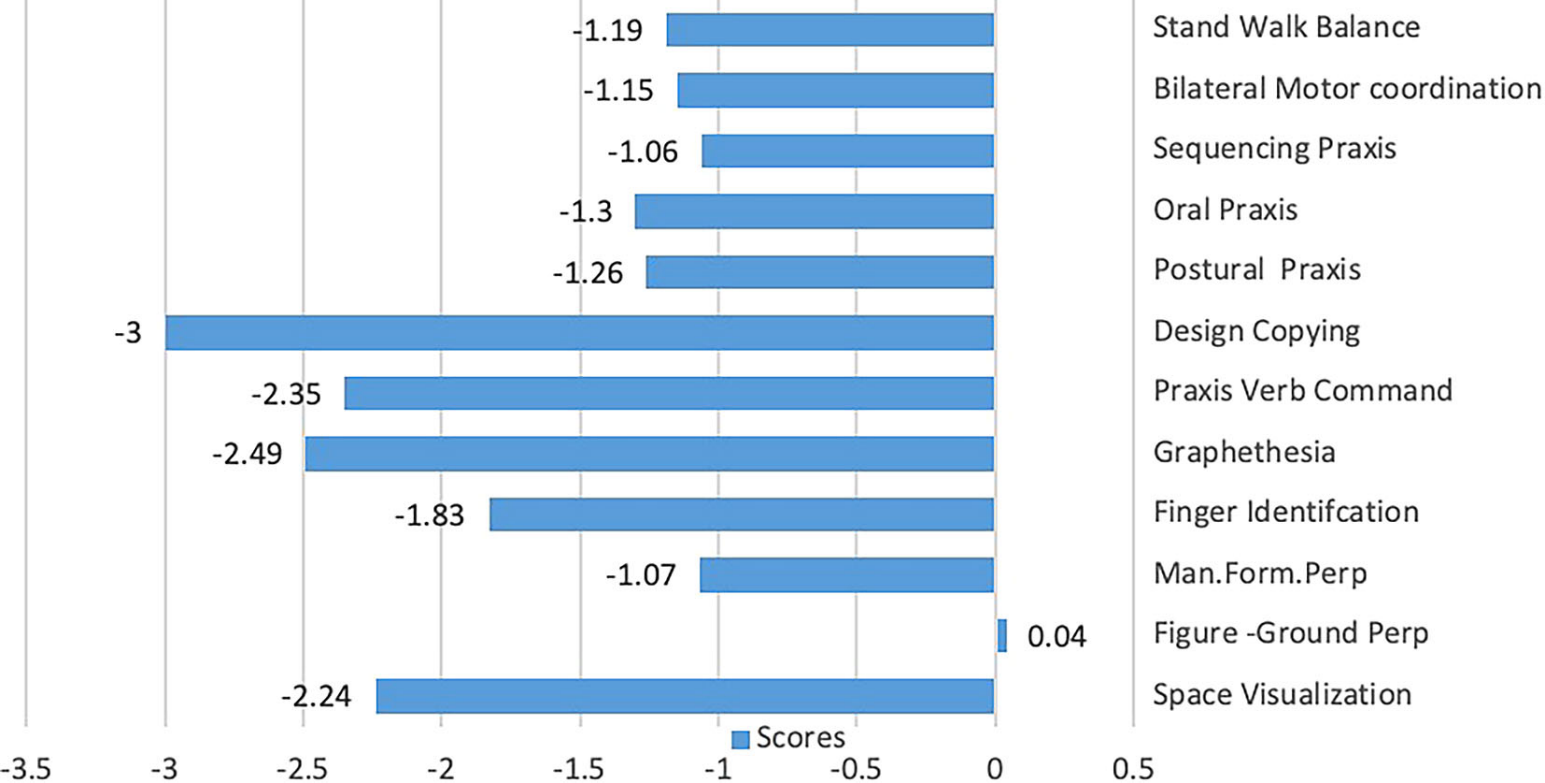
L’s Sensory Integration and Praxis Tests scores.

Results from pre-test SIPT showed that L attempted 13 of the 17 SIPTs, completing 12 (see Figure 1). Scores indicated difficulty with tactile and kinaesthetic processing, especially manual form perception, finger identification, and graphesthesia. L struggled with motor planning as measured by postural praxis, oral praxis, sequencing praxis, design copy, and bilateral motor coordination. Similarly, she scored low for space visualization. L was unable to participate in the localization of tactile stimuli, kinesthesia, and constructional praxis tests, and was unable to fully participate in motor accuracy and post-rotary nystagmus tests. L performed very poorly on tests of both fine and gross motor skills on the PDMS-2, and her total motor quotient score was very poor ( Table 1).

**Table 1.** Pre- and post-intervention Peabody Developmental Motor Scale-2 (PDMS-2) scores.

Variable	Pre-test			Post-test		
	Quotient	Standard Score	Percentile	Quotient	Standard Score	Percentile
Fine Motor	64	8	1	85	15	16
Gross Motor	70	16	5	91	26	27
Total Motor	64	24	1	88	41	21

The findings from the Arabic SPM-P classroom and home forms are illustrated in Table 2 and Table 3, respectively. The environmental difference score demonstrated consistency across environments. Both measures indicated that L struggled with balance and motion, social participation, hearing, and touch. The home test suggested definite dysfunction for almost all scales, including social participation, hearing, body awareness, balance and motion, planning and ideation. Except for vision which was typical, the remaining two scales, touch and total sensory system, also ranked high, indicating that L had difficulties in those areas as well. In comparison, the school form suggested less dysfunction, with only the hearing scale ranked as definitive dysfunction, while the rest fell between typical and problematic. The lack of significant correlation between the home and classroom results might originate from the divergence between adult expectations and daily routines at home as opposed to school (Parham *et al.*, 2007). Her mother’s expectations were definitely higher for how she acted at home, such as her clumsy movement, her unwillingness to wear or put on shoes, her utilise and grade appropriate force when she holds writing and other tools, and her tendency to bump into others and the results supported that since they were almost entirely in the definitive dysfunction ranking. On the other hand, the lack of correlation could relate to the Saudi culture, as parents tend to overprotect their children. This attitude could limit ASD children’s opportunities to be stimulated physically and, thus, to process proprioceptive and vestibular information ( Al-Heizan *et al.*, 2015).

**Table 2.** Pre- and post-intervention Arabic Sensory Processing Measure-Preschool (SPM-P) school form scale scores.

Variable	Pre-test				Post-test			
	Raw score	T-score	%ile rank	Functioning	Raw score	T-score	%ile rank	Functioning
Social participation	31	80	99	SP	19	54	66	T
Vision	17	58	79	T	10	40	16	T
Hearing	20	70	98	DD	12	53	62	T
Touch	18	68	62	SP	12	56	66	T
Body awareness	20	74	99	T	12	56	69	T
Balance & motion	22	72	99	SP	11	53	62	T
Planning & ideation	27	77	99	SP	13	54	66	T
Total sensory system	107	69	97	SP	64	51	54	T

**Table 3.** Pre and post intervention SPM-P home form scale scores.

Variable	Pre-test				Post-test			
	Raw score	T-score	%ile rank	Functioning	Raw score	T-score	%ile rank	Functioning
Social participation	26	66	95	DD	10	44	27	T
Vision	12	51	54	T	17	58	79	T
Hearing	22	74	99	DD	14	60	84	SP
Touch	16	66	95	SP	16	46	34	T
Body awareness	12	55	73	DD	11	50	50	T
Balance & motion	14	64	92	DD	13	55	69	T
Planning & ideation	20	66	95	DD	9	40	16	T
Total sensory system	82	62	88	SP	78	56	73	T

*Note.* SP = Some Problems; T = Typical; DD = Definite Dysfunction.

### Hypotheses and individual goals

In line with the theory of ASI and the practice guidelines for OT, the goals were based on the assessment findings and concentrated on the underlying conditions that impacted L’s development. Overall, the findings suggested L’s participation challenges were due to impairments related to SI and praxis. Specifically, it was hypothesized that L had somatodyspraxia ( Schaaf & Mailloux, 2015).

The first author worked with L’s mother to identify specific and measurable goals reflecting L’s functional challenges ( Schaaf & Mailloux, 2015). L’s mother knew about her difficulties in school and could contribute to setting both school- and home-based goals. As recommended by Ruble *et al.* (2012), goals were quality checked by the second author.

The goals were established using the Goal Attainment Scale (GAS; King’s College London) ( Ruble *et al.*, 2012). The GAS is both systematic and sensitive to changes in functioning associated with ASI. It entails describing the individual’s present level of functioning for a given goal and then scaling it for the anticipated level of function over the intervention period. GAS helps assess outcomes that are challenging to measure with traditional instruments ( Mailloux *et al.*, 2007), and is a valid and reliable tool ( Steenbeek *et al.*, 2007) for individuals with ASD ( Ruble *et al.*, 2012). GAS uses a five-point scale, with values ranging from -2 to 2, scaled with equally spaced probability intervals ( Kiresuk *et al.*, 2014). A score of 0 reflects the anticipated level of function, while -1 and -2 suggest a level of attainment less/much less than anticipated, respectively, while +1 and +2 suggest a level of attainment better/much better than anticipated, respectively. Scores for each goal are used to calculate T-scores, representing the extent to which functioning improved to the degree anticipated.

L’s goals were:

1. *Dressing*: Improve participation in dressing, to independently put on shoes after verbal prompts with fewer than two physical prompts.
2. *Fine motor skills*: Improve participation in learning activities, to draw or reproduce a circle with two verbal prompts.
3. *Play*: Improve participation in social play, engaging with a peer in age-appropriate activities for 10 minutes with two adult redirections.
4. *Safety:* Improve participation in playtime, navigating the playground without bumping into objects or people.

These goals were based on the premise that improving sensory processing would increase participation in everyday activities ( Schaaf & Mailloux, 2015).

The authors determined the proximal and distal outcomes to use to track progress *.*

*Distal Outcome Measure:* Changes in L’s goals were measured by GAS, enabling the evaluation of progress toward specific, measurable, and time-dependent goals.

*Proximal Outcome Measures:* The Arabic SPM-P and the PDMS were used as secondary outcome measures. The former was used to measure sensory reactivity, praxis, and social participation, the latter to measure fine and gross motor skills.

### Intervention

The intervention was designed with the approval of L’s mother. L would participate in 1-hour ASI sessions twice weekly for 10 weeks. Meanwhile, the author would meet her mother in person and have discussions with her teacher over the phone for 30 minutes every week. Each session would provide sensory-rich experiences to elicit changes in L’s behavior. Accordingly, L’s sessions were designed to provide a challenge level that was “just right” for her sensory systems ( Schaaf & Lane, 2015).

For therapy room sessions, the intervention was divided into three phases: early, intermediate, and final sessions ( Schaaf & Davies, 2010). Schaaf and Davies (2010) emphasized that ASI entails attention to meaningful activity, requiring adaptive responses and active participation from the child. L had numerous opportunities to play with tactile-rich apparatus, to improve her proprioceptive and tactile perception, and praxis. Moreover, through pulling, pushing, and hanging activities, she was encouraged to stretch and engage her muscles. She participated in various active sensory–motor activities. She used a scooter board to experience proprioceptive and vestibular sensations and increase body awareness. She also engaged in jumping into a ball pit, climbing, rolling, and crawling. There were opportunities to change the apparatus and the rhythm, duration, frequency, and/or intensity of sensory experiences, based on L’s responses. In line with ASI principles, the first author and child cultivated an active, trusting relationship, with the former monitoring activity demands to ensure a just-right level of challenge ( Schaaf & Mailloux, 2015).

The use of the ASI Fidelity Measure also ensured the intervention was in line with ASI principles ( Parham *et al.*, 2011). This measure defines the structure of the intervention and process elements ( Parham *et al.*, 2011; Schaaf & Mailloux, 2015). A score of 80 is a tentative cut-off point for adherence to ASI ( Parham *et al.*, 2011). The measure has high interrater reliability for both total fidelity scores (.98) and individual items (.94–.99). The validity of the measure is strong as raters can distinguish ASI sessions from other interventions with 92% accuracy ( Parham *et al.*, 2011).

All 20 sessions were videotaped, and the second author, trained in the application of the measure and with 30 years of experience working with children with ASD, assessed four randomly selected tapes. Additionally, the second author provided weekly consultations to the first author and was available throughout to discuss intervention challenges. Only one session produced an unacceptable fidelity score, after which the authors collaborated to ensure acceptable fidelity in all other sessions. The mean fidelity score across the four sessions assessed was 85 ( *SD* = 9).

The early sessions focused on getting L accustomed to the environment, therapist, and equipment on the playground. The author worked with L to help her understand that she could make her own choices. L also learned that she had her own space that she could explore and enjoy. Engaging her in a funny way also provided the therapist more possibilities to gauge her moving abilities. The first sessions gave L many opportunities to develop and use proprioception and vestibular and tactile sensations. The OT notes revealed that L would often choose a familiar object and avoid motor activities. She would always rely on the therapist to initiate activities and presented a low level of adaptive response. Transitions proved to be a real challenge and she needed a lot of positive feedback from the therapist to proceed with new sensory experiences. The therapist learned how to scaffold the activities so that L would get the right challenge and she moved the equipment around to adjust it to L’s needs.

During the intermediate sessions, L became more self-confident and able to express herself, although she needed continuous feedback and encouragement. She was also more confident in her therapist and engaged in the tasks with much more ease than before. The therapist learned to be more specific about the feedback and presented more sensory opportunities, such as tactile and challenge praxis. Under careful guidance and encouragement from the first author, L finally got on the swing, which was acknowledged and celebrated by L’s mother, teacher, and therapist. L was provided with additional tasks that would involve the rotary vestibular eye tracking and crash-bang-or-jump activities.

Over the last sessions, the therapist strategically created specific themes for each session to develop L’s ideation and planning. She also continued to provide L with proprioceptive, vestibular, and tactile sensations for successful integration. L started making choices, initiating, and participating with far fewer prompts. She was able to sustain and develop individual activities for a much longer period. Moreover, she did not feel the urge to withdraw when new activities were introduced to her. She was much more confident in her abilities to engage with the tasks. During the last sessions, L was slightly fatigued with a virus and then a sandstorm hit the region. Her improvements were especially significant in this last phase since the virus and the weather could not stop her from enjoying the sessions and showing obvious progress.

## Results

Outcomes were measured consistently to monitor progress during the intervention. The post-intervention results were measured by repeating the SPM-P, GAS, and PDMS-2 tests after two weeks of the last session. Clinical observations noted the improvements L made over the intervention period. The ratings of L’s mother and teacher for each goal were added to calculate a T-score using Kiresuk *et al.*’s (2014) formula. L’s post-intervention T-score was 62, suggesting she performed better than expected.

**Figure 2.**
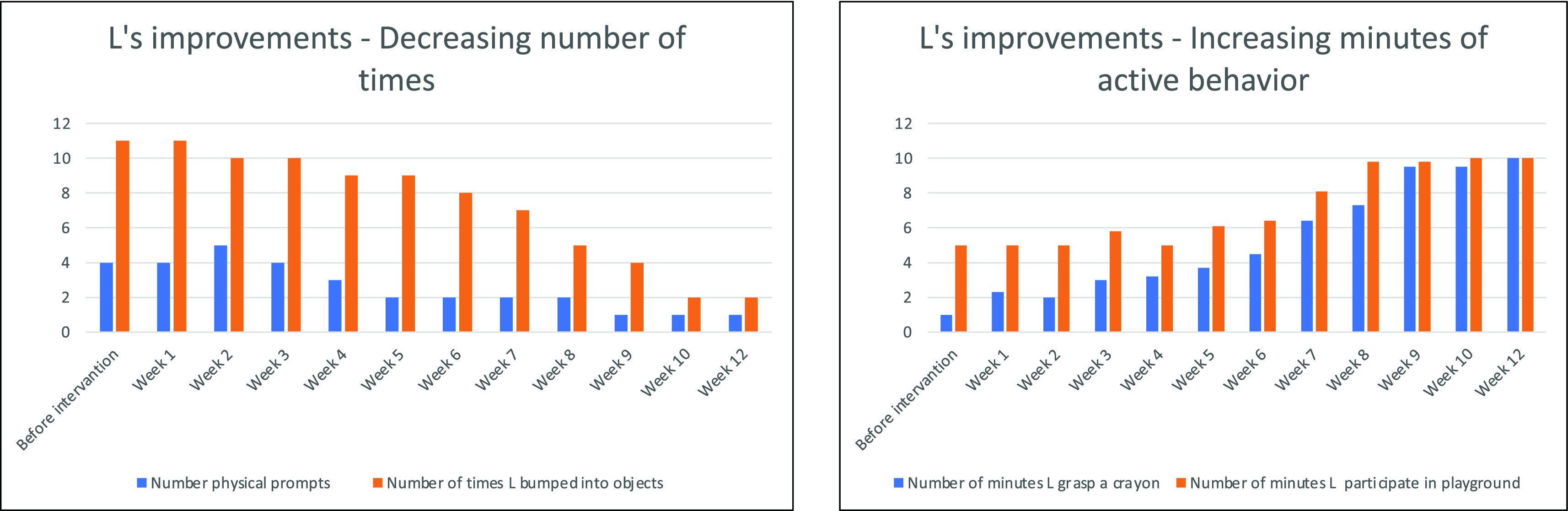
Weekly data collection sheets.

The findings showed that L improved in various domains. The GAS scores showed that outcomes for the dressing goal (rating = 1) exceeded expectations. Outcomes for the other goals (ratings = 0) were consistent with expectations. Since two of the goals were oriented towards skill acquisition (dressing and drawing), and the other two were concerned with behavioral reduction (bumping and avoid playing), the results are presented separately for each goal. The skills consistently improve and the problematic behavior decreased over the ten-week intervention, as the goal charts show ( Figure 2).

**Figure 3.**
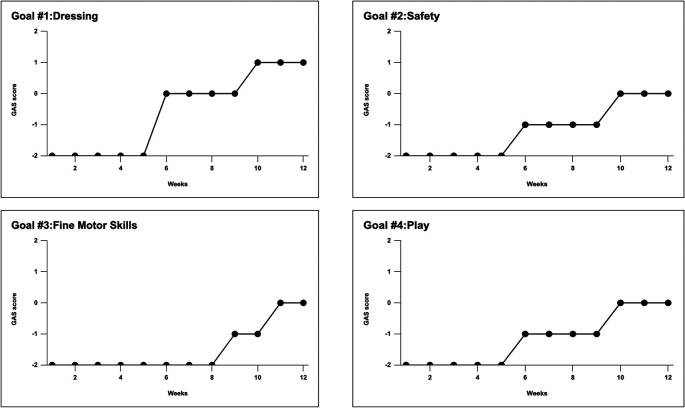
Goals and goal attainment scales. L demonstrated visible improvements in all goals.

Visual inspection of L’s goal attainment scales (using Excel line graphs) shows that L attained her target level in each of the four OT goals as depicted in Figure 3. To summarize, the first goal was related to dressing and stated, “After initial verbal instruction, L will independently put on her shoes with fewer than two physical prompts.” L exceeded the previous expectations, as the outcome proves. She was able to put on her shoes with just one physical prompt at the end of the intervention (+1). The second goal was about safety, “L will be able to navigate the playground safely during break without bumping into objects or people during the school or home.” L managed to navigate the playground with no more than two physical cues at the end of treatment (0). The third goal regarded motor skills: “L will demonstrate age-appropriate fine-motor skills as a basis for participation in school activities.” By the end of the last sessions, L was able to draw using the crayon appropriately for 10 minutes without interruptions (0). The fourth goal focused on playing: “L will play with a peer in an age-appropriate activity for 10 minutes.” L reached this goal also she was able to play for 10 minutes without interruptions or fussy behavior.

The results for the Arabic SPM-P home and classroom show L improved in sensory reactivity, praxis, and social participation in classroom ( Table 2) and home settings ( Table 3). Similarly, the PDMS-2 data indicated L’s overall motor skills improved ( Table 1). As Table 2 and Table 3 show, the scores of post-intervention SPM-P classroom and home assessments improved substantially. While hearing still presented some problems, the percentile rank for the rest of the items fell into the typical range in the home test. The items were no longer rated problematic or dysfunctional in the school test, showing more consistency with the home test than the pre-intervention assessments.

PDMS-2 scores ( Table 1) showed that L’s motor skills improved during the intervention, from 64 to 85 for fine motor, 70 to 91 for gross motor quotient, and 64 to 88 for total motor quotient. These scores were in line with SPM-P findings and the therapist also noted the positive motor improvements L made over the ten treatment weeks.

The OT observations also revealed improvements ( Figure 2). The author compared L’s goal attainment rating across three time points (before, midst, and after intervention) and observed a continuous improvement, 80% of the time as observed by the therapist and reported by mother and the teacher. For example, the number of physical prompts for L to put on her shoes decreased from four to one during the intervention. Also, bumping into objects decreased from 11 before the ASI intervention to 2 after the intervention. On the other hand, the time she spent drawing or participating in the playground increased over the span of the intervention from a couple of minutes to ten for both drawing and playing goals.

The author also conducted a second interview with L’s mother and teacher six weeks after the intervention. L’s mother reported that, despite receiving no additional sessions, L’s behavior at home and school continued to improve. L’s mother described her as happier, more sociable, more able to play safely, and less impulsive. She said:

“ *L has started to play more. Yesterday, she created a restaurant menu and when she was upset, she drew a sad face! She can sit and play for more than 20 minutes. In addition, she hugs me without using too much force. L does not react to unfamiliar tasks with crying anymore! I wish many centres could offer the ASI … L cannot wait for your sessions*!”

When interviewed six weeks post-intervention, L’s teacher said that her *“attention in the classroom was so much better and she is starting to participate in making choices during activities.*” Both reported that L’s fine motor and social skills had improved, and she showed fewer difficulties when playing.

Post-intervention, L performed better on sensory and motor tasks and, in the context of ADLs, she demonstrated improvements in fine, gross, and overall motor skills. Previously, her performance was characterized as “very poor”, subsequently it was “average” or “below average”. These findings are in line with the review of Schaaf *et al.* (2018).

Jasmin *et al.* (2009) found that sensorimotor deficits in those with ASD underlie their day-to-day functioning. Congruent with this, L’s improvements in sensory processing and motor skills were reflected in enhanced performance in ADLs. For all tasks identified by GAS, she exhibited increased ability. She improved, as much as anticipated, her capacities to draw, play appropriately, and dress herself, positive changes that were reflected in the comments of L’s mother and teacher as well. L also started to participate in making choices during the activities and showed improvements in her concentration. The Figures 2 and 3 shows L’s progress from the first baseline phase through the intervention to the last baseline phase. Finally, in a conversation six months post-intervention, L’s mother reported that, despite receiving no additional sessions, L’s behaviour at home and school continued to improve.

## Discussion

The discussion about ASI application in SA requires some remarks regarding the contextual factors influencing the treatment and the outcomes of the study. According to Serbin *et al.* (2014), a child’s family is the main influence on their health and well-being. However, the parents of disabled children are especially susceptible to stress ( Dempsey *et al.*, 2009), potentially limiting their contributions to GAS. Indeed, L’s mother struggled to imagine goals that would reflect better regulation and participation in family life. Moreover, Saudi mothers often place great trust in healthcare providers and may feel it inappropriate to engage in the therapeutic process ( Alkhalifah & Aldhalaan, 2018); this appeared true of L’s mother, who was hesitant to participate in GAS. Nonetheless, this study suggests that GAS is useful for quantifying individual outcomes ( Mailloux *et al.*, 2007). GAS also enables researchers to measure changes in tailored, functional, and parent-generated goals. Therefore, it is a valuable supplement to other assessments when measuring outcomes associated with individual interventions ( Schaaf & Mailloux, 2015). The use of proximal and distal goals was helpful in demonstrating links between the underlying challenges in SI and daily occupation.

The SIPTs had not previously been applied in an Arab nation, so this was the first exploration of their suitability in SA. However, some SIPTs can be difficult to administer to non-English-speaking children ( Bodison & Mailloux, 2006). Roley *et al.* (2015) highlighted that SI tests can be challenging for children with ASD, with only 63% of their sample finishing most tests. Therefore, proxy measures (e.g., observations) are recommended ( Schaaf & Mailloux, 2015). Other limitations of the SIPTs are that they rely on 40-year-old normative data and their software is incompatible with modern operating systems ( Mailloux *et al.*, 2018). The SIPTs are also only suitable as pre-post-test measures for periods exceeding eight months ( Mailloux *et al.*, 2018). In the current study, L could not complete all the SIPTs; therefore, additional measures were utilized, with GAS completed post-intervention.

Treatment integrity was confirmed using the Ayres Sensory Integration ^®^ Fidelity Measure ( Parham *et al.*, 2011). This presented some challenges. For instance, it was necessary to videotape the sessions. While L’s mother agreed to this, many Saudi families may not, due to stigma and religious reasons ( Alotaibi & Almalki, 2016). L’s mother expressed concerns and wanted to ensure no one beyond the first author and the training group would see the video; she personally refused to be videoed, or audio recorded, for religious reasons. Furthermore, accessing suitable training was difficult.

In light of the above hindrances, the study was successful in terms of gathering data about the patient, implementing the intervention, and measuring the outcomes with the few measuring instruments available at that time, and also heeding to the cultural, religious, and societal restraints. The findings are very significant for future research endeavours in SA because they demonstrate how ASI interventions can be conducted even in the absence of measuring instruments translated into Arabic or adapted to this specific context. The findings informed Saudi therapists on several effective treatments and scales that have not been administered in conjunction with each other before in SA. This was the first exploration of the suitability of tests like SIPT or GAS in SA. GAS measured the outcomes for L’s fine and gross motor skills.

The results show consistency across various tests from pre to post intervention. The tests used in the assessment period (SIPT, Arabic SPM-P, and PDMS-2) converged towards the hypothesized diagnosis that L had somatodyspraxia. Interview data gathered about L’s participation in daily activities as reported by L’s mother and the school teacher were congruous with the test results about poor sensory processing and motor skills. The SPM-P and PDMS-2 were conducted again post-intervention and GAS was added to probe the reliability of results. The scores indicated L reached the goals considered typical for kids of her age regarding safety, fine motor skills, and playing. She even exceeded expectations for dressing, one of the areas that was rated difficult before. The second interview with L’s mother and the teacher revealed that the progress was obvious and confirmed once again the findings of the assessment tools.

We also want to stress here the importance of clinical observations for providing insights of a qualitative nature. This study pioneered a clinical treatment and utilized several measuring instruments that had never been used together before in the Saudi context. Although the clinical observations were unstructured, they contributed substantially to mapping the child’s behavior, performance, improvements, and outcomes. The scores show the quantitative difference between phases, but the observations translated those findings qualitatively so that Saudi therapists can see all the details behind the figures and numbers. The Fidelity scores show that the author abided by the standards of the intervention, but the clinical observations show how she was able to reach those standards. This behind-the-scenes perspective enhances the case report evidence provided by this study.

### Preliminary evidence for the efficacy of ASI in SA

Given the lack of research describing ASI in SA, this case study examined the feasibility and effectiveness of this intervention. The study suggests ASI is feasible within SA and can lead to improvements in individualized functional goals in ADL and performance on sensory and motor tasks. Not only is the ASI intervention feasible, but the measuring instruments also proved reliable in this context.

However, not all tests measuring SI are suitable in the SA context. The SIPTs had not previously been applied in an Arab nation and they fared questionably in the first exploration of their suitability. In the current study, L could not complete all of the SIPTs; therefore, additional measures were utilized, with GAS completed post-intervention. GAS provided more accurate results and was more effective for quantifying L’s progress during the intervention. SPM-P and PDMS-2 scales could also be implemented to measure ASI interventions in SA and the Fidelity scores demonstrated that therapists could use such instruments with confidence in this socio-cultural context.

The therapist’s unstructured notes and clinical observations as well as feedback from L’s mother and teacher were discussed at length to offer more evidence for ASI implementation challenges and opportunities in SA. The detailed presentation and analysis of data offer support for efficient ASI implementation in ASD children from SA in the future. The authors ensured that the experiment was fully video-recorded and the tools available were rigorously assessed.

Our recommendation for future related studies is to utilize the Evaluation of Ayres Sensory Integration (EASI) instrument, released in 2020. Suitably qualified therapists have open access to the EASI, which incorporates normative data collected internationally. The EASI meets the demand for the assessment of ASI constructs with psychometrically validated and internationally applicable measurement tools ( Mailloux *et al.*, 2018).

### Limitations

The limitations of the study are divided into two categories: (1) research-related challenges, and (2) cultural context. The authors did not have access to data regarding previous ADOS diagnosis. L was diagnosed with ASD level 2, at 2 years and 3 months by a multidisciplinary team, but the data were not available. The study adopted an experimental single case report of one Saudi child; therefore, the extent to which the results can be generalized is limited, even in the context of SA. Future studies in SA could replicate this study using the measuring instruments used here. Other tools may render different results because ASI interventions have not been regulated yet in Saudi Arabia. The lack of resources translated into Arabic plays a very significant role in the limitations of this study, as discussed below. Another limitation is that the evaluation was completed by the therapist delivering the treatment, not independent assessors, as that was not possible.

Neuroscientific evidence suggests OT-ASI interventions elicit increased neurological adaptations if they simultaneously target multiple sensory systems ( Lane & Schaaf, 2010). However, acquiring apparatus suitable for stimulating L’s various sensory systems was challenging; few items were available in SA, so they had to be ordered internationally. The ASI fidelity tool requires a suitable room, points of suspension, and equipment; negotiating additional space and financing for this was time-consuming.

However, most limitations were connected to the lack of resources in Arabic and the shortage of teachers and qualified therapists trained in ASD interventions. A few Arabic language resources are available, such as a translation of the SPM-P ( Alkhalifah *et al.*, 2020). It is necessary to translate more resources into Arabic to increase understanding of ASD and SI among teachers in SA, to inform their practice and help them identify pupils who might benefit from OT interventions. While OT-ASI in SA appears feasible, its delivery was not straightforward. Since OT-ASI has only recently been introduced to SA, few parents were aware of its potential, making it challenging to find willing participants. This was partially addressed by translating materials that explained what ASI is and why it is valuable, which was a labour-intensive process.

Teachers in SA rarely participate in interventions and have limited knowledge of ASD and sensory issues ( Haimour & Obaidat, 2013). Therefore, the first author spent time explaining to L’s teacher how to complete the Arabic SPM-P and was available to answer questions. Additionally, the first author was available to provide L’s mother and teacher with guidance to understand the intervention. However, therapists in SA working with individuals with ASD and their families often lack time to model interventions or address families’ complex medical and behavioral needs ( Alnemary *et al.*, 2016).

Some Saudi professionals do not share alternative treatments with families because of the limited hands-on support available for ASD interventions ( Alshehri *et al.*, 2019). This shortage of qualified trainers could reduce the quality of services in SA for ASD ( Alnemary *et al.*, 2016). The first author undertook training abroad for over one year. To complete this study, the therapist required confidence and experience in navigating the local protocols and ethical consent processes ( Schaaf & Nightlinger, 2007).

## Conclusion

This study hypothesized that participation challenges observed in a child with ASD were linked to SI impairments. In support of this, improvements in her participation were seen after an AS intervention targeting SI. Therefore, this study provides initial evidence that Saudi children with ASD could benefit from ASI treatments and offers insights into the factors affecting delivery.

This study highlights the lack of normative data on SI in SA and a need for further Arabic assessment tools. Moreover, there is little awareness of how individuals with ASD face challenges in SI, and relevant resources are insufficient. The government could address these issues through ASD and ASI workshops, establishing scholarships for training, and committing financial resources to assessment tools and treatment spaces.

Creating videos was particularly valuable as the child’s responses could be reviewed, helping the therapist to reflect. Similarly, the intervention was facilitated by DDDM (Schaaf & Blanche, 2012). Post-graduate training in ASI and GAS supports service delivery and the evaluation of outcomes and mentored training is recommended for newly qualified OTs. Nevertheless, this study provides a stepping-stone to further research in this area for OTs in SA working with children with ASD whose functioning is impacted by aberrant SI.

### Key points for occupational therapy

• There is a need for more data on SI in SA and Arabic assessment tools.
• Government-funded workshops, training scholarships, assessment tools and treatment spaces would help address challenges in SA.
• Post-graduate and mentored training in ASI and GAS are recommended for newly qualified OTs.

### Ethics and consent

The study was presented to the Centre for Autism Research (CFAR) at King Faisal Hospital and ethical approval was granted for it to proceed. L’s mother gave written informed consent for L to be involved in the study and for L’s information to be published in this manuscript. L’s mother also gave permission for L to be recorded during her sessions. All the recorded sessions are stored in the CFAR’s system, in a separate, secure (locked) folder to ensure data protection. Further, in accordance with the research data management policy, all recorded sessions have been secured in password protected files only accessible to the researchers. The videos will be retained for 10 years, before being securely disposed.

## Data availability

All data underlying the results are available as part of the article and no additional source data are required.
