# Supplementary material for: Case Report: ASI intervention on a child with autism in Saudi Arabia
Source: F1000Res. 2022 May 4;11:50. Originally published 2022 Jan 17. [Version 2] doi: 10.12688/f1000research.74257.2 (PMC9326240; doi:10.12688/f1000research.74257.2)
Supplement: Peer review history

# Peer review: Reviewer response for version 2

**DOI:** 10.5256/f1000research.132424.r142911

**Published:** 2022-07-26

**Authors:** Sana M. N. Abu-Dahab

**Affiliations:** 1 Department of Occupational Therapy, School of Rehabilitation Sciences, The University of Jordan, Amman, Jordan

Dear Authors,

 Thank you for this opportunity to review your paper. Such studies are lacking and highly needed in the Arab region.

 Consider the following changes:

1. You have used the terms sensory integration (SI) and sensory processing (SP) interchangeably, however, there is a slight distinction. SP refers to the changes/disorder itself in processing sensory information while SI is the frame of reference for understanding, assessment, and intervention of SP. I suggest looking into Miller *et al*. 2007 ^1^ and adjusting the introduction accordingly as you refer to the disorder itself as SP and the intervention as the SI or Ayres SI or OT-SI.
2. In paragraph 3 in the introduction – what do you mean by “manual” ASI? Please clarify.

**Methods**

1. Start with describing your participant. Your procedure should come last after describing your participants and your outcomes - please bring all the information regarding L (then you have before “assessment” at the begging and this would give the reader a better understanding of the intervention).
2. Did L have an OT before even not OT-SI?
3. You mention that L's “cognitive development was typical”, as measured by what? Or as an indication of what?

**SPM**

1. Would you further explain the scoring of SPM. Especially when it comes to percentile and functioning. Usually, higher percentiles are indicatives of better performance, but as my understanding from the table, this is not the case.

**GAS**

1. Please provide a GAS table of your -2 -1 0 1 2 grading of goals. Using GAS is an excellent choice which is being more practiced recently. As L started at -1, it would be of interest to the reader to see the whole grading of the goal.
2. You mentioned before the results, “virus and the weather could not stop her from enjoying sessions”, would you clarify what you mean by “enjoyment”? How did L communicate enjoyment to you? This is also of significant importance to the reader and clinicians.

Are enough details provided of any physical examination and diagnostic tests, treatment given and outcomes?

Partly

Is the case presented with sufficient detail to be useful for other practitioners?

Partly

Is sufficient discussion included of the importance of the findings and their relevance to future understanding of disease processes, diagnosis or treatment?

Yes

Is the background of the case’s history and progression described in sufficient detail?

Yes

Reviewer Expertise:

Developmental Disabilities/Occupational Therapy

I confirm that I have read this submission and believe that I have an appropriate level of expertise to confirm that it is of an acceptable scientific standard, however I have significant reservations, as outlined above.

## References

[1] : Concept Evolution in Sensory Integration: A Proposed Nosology for Diagnosis. *The American Journal of Occupational Therapy* .2007;61(2) : 10.5014/ajot.61.2.135 135-140 10.5014/ajot.61.2.135 17436834

# Peer review: Reviewer response for version 2

**DOI:** 10.5256/f1000research.132424.r136703

**Published:** 2022-05-05

**Authors:** Sayyed Ali Samadi

**Affiliations:** 1 Institute of Nursing and Health Research, Ulster University, Coleraine, UK; 2 Bahoz Center for Children with Developmental Disabilities, Erbil, Iraq

The revised version is an improved version of the previously reviewed manuscript. I generally track my previous comment in the text in any second review and follow my recommendations to see if the raised issues are addressed or not. In the present format, it was not possible. Therefore, I re-reviewed the study. This version is a real improvement. The text is more focused, and especially the limitation section is more informative from a methodological aspect.

Are enough details provided of any physical examination and diagnostic tests, treatment given and outcomes?

Yes

Is the case presented with sufficient detail to be useful for other practitioners?

Partly

Is sufficient discussion included of the importance of the findings and their relevance to future understanding of disease processes, diagnosis or treatment?

Partly

Is the background of the case’s history and progression described in sufficient detail?

Partly

Reviewer Expertise:

Intellectual and developmental disabilities

I confirm that I have read this submission and believe that I have an appropriate level of expertise to confirm that it is of an acceptable scientific standard.

# Peer review: Reviewer response for version 1

**DOI:** 10.5256/f1000research.77990.r124289

**Published:** 2022-02-28

**Authors:** Gustavo A. Reinoso

**Affiliations:** 1 Department of Occupational Therapy, Nova Southeastern University, Clearwater, FL, USA

Please see my comments below:

 Case reports are very important to build evidence based practice. I would suggest some changes to the structure and how data were presented. The authors may find case report standards (e.g. https://www.care-statement.org/) useful in structuring the report. The manuscript would also benefit from establishing a more fluid connection between a) clinical findings, b) outcome measures, and c) GAS goals. Similarly, a more comprehensive description of the intervention (what was done) and how fidelity was achieved may also enhance your case. I am also adding a few recommendations below. Thank you for your work and contribution. 

1. Discuss the limitations of using the SIPT. This is a 33 year old test with unknown parameters in other countries such as SA.
2. Spell check "heigh reliability."
3. Validity and reliability are NOT properties of a test (they are parameters that refer to a specific sample. Thus, the parameters listed do not directly apply to your study). *"Moreover, the test has high inter-rater reliability due to the detailed scoring and because therapists using the SIPTs undertake training (Schaaf & Mailloux, 2015). Each SIPT results in a standard score that reflects the child’s performance compared to age-matched norms; the average score for a group of a given age is 0 (Schaaf & Mailloux, 2015). Scores below -1 standard deviation are considered evidence of dysfunction."*
4. Use primary references when discussing assessment tools (e.g. SPM and  (Jorquera-Cabrera *et al.*, 2017).
5. Spelling "kinesthesis" vs Kinesthesia
6. SIPT Scoring: Provide a qualitative summary of her overall performance during testing (e.g. organization of behavior, difficulties following directions, ability to imitate/copy, understanding instructions, etc). This will be more useful than scores derived by comparing your case with an older American reference sample.
7. Describe why unstructured observations were not completed if the child presented with difficulties with imitation/copying, following directions, etc. or provide a summary of how they were administered, scored, etc. " *Additionally, on clinical observation, L was unable to maintain a flexion position for more than 10 seconds, while prone extension was achieved for 3 seconds. She showed difficulty with sequential finger touching and ramped arm movements. She also avoided touch in the absence of vision"*
8. *Step VI * - Consider rephrasing: "... in the aberrant SI hypothesized'
9. Reconsider using a questionnaire as a post test measure.

Are enough details provided of any physical examination and diagnostic tests, treatment given and outcomes?

Partly

Is the case presented with sufficient detail to be useful for other practitioners?

No

Is sufficient discussion included of the importance of the findings and their relevance to future understanding of disease processes, diagnosis or treatment?

Partly

Is the background of the case’s history and progression described in sufficient detail?

Yes

Reviewer Expertise:

Children and youth, disability, sensory processing, sensory integration, handwriting, measurement and statistics, Rasch analysis, assessment tool development.

I confirm that I have read this submission and believe that I have an appropriate level of expertise to confirm that it is of an acceptable scientific standard, however I have significant reservations, as outlined above.

# Response

**Published:** 2022-04-17

**Authors:** Shahad alkhalifah

**Affiliations:** King Faisal Specialist Hopital and Research Center, Saudi Arabia

Dear Dr. Gustavo,

 We are pleased to submit a revision of our manuscript entitled: “ **Case Report: ASI intervention on a child with autism in Saudi Arabia**” for publication in F1000Research.

 We have revised it to take into account your valuable comments. Thank you for these appreciative comments; we now addressed all your comments and feedback.

 We are looking forward to receiving an answer from you at your earliest convenience.

 Sincerely,

 Shahad Alkhalifah

# Peer review: Reviewer response for version 1

**DOI:** 10.5256/f1000research.77990.r120181

**Published:** 2022-02-07

**Authors:** Sayyed Ali Samadi

**Affiliations:** 1 Institute of Nursing and Health Research, Ulster University, Coleraine, UK; 2 Bahoz Center for Children with Developmental Disabilities, Erbil, Iraq

This study is important from the perspective of the cultural aspects of autism spectrum disorders (ASD). It is important because of the shortage of available knowledge on the applicability of the Western data, scales, and ASD protocols on non-Western cultures.

 The manuscript presented some justification on the importance of considering sensory issues for individuals with ASD and reconsidered the sensory issue in the context of Saudi Arabia. The main aim, which is attainable through meeting two objectives, is cited at the end of the introduction part under a sub-heading. This study has aims and objectives which are clearly indicated and although this is a case report, it needs a part allocated to the methodology - this is important. The primary goal of the case study is to give readers an opportunity to see how the guidelines apply in the context of an actual situation (i.e. SI as an applicable approach for the Saudi individuals with ASD). The study methodology is the specific approach or technique that is used to identify, select, process, and analyze information about a topic and allows the reader to critically evaluate a study's overall validity and reliability. It is very helpful to understand why a case report is selected to answer such important research questions. Which design of the single case study was adopted? What is the logic behind the selection? All this information shapes a paragraph that is very helpful to understanding the study.

 The other issue is mentioning ADOS, but not ADOS-2 (Lord *et al*., 2012 ^1^, Pruette, 2013 ^2^) as an evaluation for diagnosis without presenting any information about the applicability of this sophisticated scale on the Saudi Arabia children with ASD population. The irony is that the study tackles the issue of applicability of intervention approaches on non-Western cultures. I think since the diagnosis has already been done by a multidisciplinary team of experts, this scale might be excluded in this part. If no data is available, it deserves to be stressed (probably in the limitations part).

 It is very important to understand how parents were approached, how did they receive information about the study? How many were approached and how many finally accepted? How many children were also excluded because of the exclusion and inclusion criteria?

 Autism implies to a very heterogeneous group of individuals and since this is a single case report, presenting some information about the level of ASD (level 1, 2, or 3 based on DSM5 ASD classification) or even the level of severity of the core symptoms of ASD will be very helpful.

 This is very interesting to see the level of application of different scales to attain goals to understand the fidelity, but I think what is missing in the findings part is the visual presentation of the attained objectives in the duration of the intervention. This is included in most of the single-subject designs. This is generally in the form of graphs showing the baseline and also impacts of the intervention in different stages of the rehabilitation process. Single-subject research, opposite to the sample recruitment approach, relies heavily on visual inspection. This means plotting individual participants’ data recorded at the end of each session, looking carefully at those data, and making judgments about whether and to what extent the independent variable (in this study SI) affected the dependent variable (which is L’s performance in each domain). The visual inspection also shows the pace of changes over time and the expected time or sessions which is needed to see the changes.

 I do not agree with the justification that is presented in the limitation part regarding the generalization of this study. The researchers intentionally adopted a single case design, therefore, they should consider all the safeguards to make sure that the finding of the design is generalizable. But if this is called a preliminary or pilot study and the authors intend to perform a study with a group of Saudi children with ASD using the ASI approach I agree that this is the limitation of the study.

 I also think that other issues need to be considered as the imitation of the study. Most of these issues have already been mentioned by the authors in the different parts of the manuscript, such as lack of trained teachers, applicable diagnostic scales, and similar issues that might impact the results.

Are enough details provided of any physical examination and diagnostic tests, treatment given and outcomes?

Yes

Is the case presented with sufficient detail to be useful for other practitioners?

Partly

Is sufficient discussion included of the importance of the findings and their relevance to future understanding of disease processes, diagnosis or treatment?

Partly

Is the background of the case’s history and progression described in sufficient detail?

Partly

Reviewer Expertise:

Intellectual and developmental disabilities

I confirm that I have read this submission and believe that I have an appropriate level of expertise to confirm that it is of an acceptable scientific standard, however I have significant reservations, as outlined above.

## References

[1] : Autism Diagnostic Observation Schedule, Second Edition (ADOS-2) Manual (Part I): Modules 1–4. *Western Psychological Services* .2012;

[2] : Autism diagnostic observation schedule-2 (ADOS-2).2013;

# Response

**Published:** 2022-04-17

**Authors:** Shahad alkhalifah

**Affiliations:** King Faisal Specialist Hopital and Research Center, Saudi Arabia

Dear Dr. Sayyed,

 We are pleased to submit a revision of our manuscript entitled: “ **Case Report: ASI intervention on a child with autism in Saudi Arabia**” for publication in F1000Research.

 We have revised it to take into account your valuable comments. Thank you for these appreciative comments; we now addressed all your comments and feedback. In the results section, we unfolded the results, adding tables and charts to offer a visual presentation of the attained goals. Moreover, we inserted a summary of the OT observations of L’s progress across the pre-assessment, intervention, and post-assessment phases.

 We are looking forward to receiving an answer from you at your earliest convenience.

 Sincerely,

 Shahad Alkhalifah
